# Assessment of the Quorum Sensing Inhibition Activity of a Non-Toxic Chitosan in an *N*-Acyl Homoserine Lactone (AHL)-Based *Escherichia coli* Biosensor

**DOI:** 10.3390/biom8030087

**Published:** 2018-09-04

**Authors:** Xiaofei Qin, Jana Emich, Francisco M. Goycoolea

**Affiliations:** 1 Institute of Plant Biology and Biotechnology, University of Münster, Schlossplatz 8, 48143 Münster, Germany; qxf200459420@163.com (X.Q.); jana.emich@wwu.de (J.E.); 2 Institute of Human Genetics, University Hospital Münster, Vesaliusweg 12-14, 48149 Münster, Germany; 3 School of Food Science and Nutrition, University of Leeds, Leeds LS2 9JT, UK

**Keywords:** chitosan, quorum sensing, antibacterial activity, quorum sensing inhibition

## Abstract

New approaches to deal with drug-resistant pathogenic bacteria are urgent. We studied the antibacterial effect of chitosans against an *Escherichia coli* quorum sensing biosensor reporter strain and selected a non-toxic chitosan to evaluate its quorum sensing (QS) inhibition activity and its effect on bacterial aggregation. To this end, chitosans of varying degree of acetylation (DA) (12 to 69%) and molecular weight (*M_w_*) (29 to 288 kDa) were studied. Only chitosans of low DA (~12%) inhibited bacterial growth, regardless of their *M_w_*. A chitosan with medium degree of polymerization (named MDP) DA30, with experimental DA 42% and *M_w_* 115 kDa was selected for further QS inhibition and scanning electron microscopy (SEM) imaging studies. MDP DA30 chitosan exhibited QS inhibition activity in an inverse dose-dependent manner (≤12.5 µg/mL). SEM images revealed that this chitosan, when added at low concentration (≤30.6 µg/mL), induced substantial bacterial aggregation, whereas at high concentration (234.3 µg/mL), it did not. Aggregation explains the QS inhibition activity as the consequence of retardation of the diffusion of *N*-acylated homoserine lactones (AHLs).

## 1. Introduction

Chitin, poly (β-(1→4)-*N*-acetyl-d-glucosamine), is the second most abundant polymer in the biosphere. It occurs in the exoskeletons of crustaceans and insects, in squid pens, the cell walls of fungi, and in diatoms, amongst other organisms. Currently, chitin is sourced at an industrial scale from shrimp and crab shell waste. Chitosan is a linear polycationic heteropolysaccharide biopolymer that is produced by partial alkaline *N*-deacetylation of chitin [1]. It is mainly composed of two kinds of β-(1→4)-linked structural units, namely 2-amino-2-deoxy-d-glucose and *N*-acetyl-amino-2-deoxy-d-glucose. However, since it is technically difficult to completely deacetylate chitin and since highly deacetylated chitosan can be re-acetylated, what is usually known as ‘chitosan’ rather refers to a family of polysaccharides with different degrees of acetylation and polymerization. Therefore, it is best to refer to this family of polymers as ‘chitosans’. The capacity of chitosans to dissolve in aqueous solutions is the commonly-accepted criterion to differentiate them from chitin. Chitosan when dissolved in dilute aqueous acid solutions behaves as a weak polybase with a pK_0_ of 6.1 ± 0.1 [2], namely in acetic, formic, succinic, lactic, and hydrochloric acid. The characteristics of a chitosan are mostly defined by its molar mass (*M_w_*) given by the degree of polymerization (DP), its degree of acetylation (DA) and its pattern of acetylation (PA). There is no precise criterion to distinguish chitosan polymers from oligomers. The DP can vary over a wide range of ~20 to ≳1000 kDa; the DA from 0 up to ~70%, and the PA from purely random to blockwise. The variation in these three parameters gives rise to a vast number of different possible chitosans.

Research on chitosans has burgeoned in recent decades due to the large number of material and bioactive properties—namely antimicrobial [3,4,5,6,7,8,9], anti-inflammatory [10], mucoadhesive [11,12], and immuno-adjuvant effects [13]—as well as the capacity to complex and condense nucleic acids [14], among others. A broad-spectrum antibacterial activity of chitosans in solution, or as nano- and microparticles or films, has been demonstrated against Gram-positive and Gram-negative bacteria [7,15,16,17,18,19,20]. The antimicrobial properties of chitosans have found applications in the food industry, as a pharmaceutical agent in the medical industry, in the textile industry, in cosmetics, in agriculture, and in wastewater purification [1,5,15]. Despite the enormous amount of research and published literature on the antibacterial and other effects of chitosan on eukaryotic cells, in general, there is lack of experimental reproducibility and often the results are discrepant. To a great deal, this is the consequence of the poor characterization or documentation of the characteristics of the used chitosan samples.

Antibiotic resistance is a serious problem that currently poses major unmet challenges and gaps in global health [21]. There are several mechanisms whereby bacteria are intrinsically resistant or acquire resistance to antibiotic treatment. They can inhibit the antibiotic’s access to drug targets, change the structure of antibiotic targets, or directly modify the antibiotics to inactivate them [21]. Nowadays, it is urgent to pursue alternative strategies for the control of bacterial infections. Quorum sensing (QS) is a form of cell density-dependent bacterial gene expression regulation mechanism controlled by a chemically exquisite signaling system that affects communal traits (e.g., biofilm formation, bioluminescence, motility, toxin production) in both Gram-negative and Gram-positive bacteria [22]. In a large number of Gram-negative species, QS is mediated by conserved small-molecule signals that act as autoinducers, termed acylated homoserine lactones (AHLs). At high cell densities, AHLs can bind to specific receptors that act as transcriptional activators of the gene cascades involved in virulence responses [22,23]. Hence, inhibition of QS can be considered a promising novel strategy to deal with bacterial pathogens at large scale while potentially circumventing the problem of antibiotic resistance development [24].

So far, the antimicrobial activity of chitosans has been extensively studied, but only limited information focuses on the effect of chitosans on bacterial QS activity. To the best of our knowledge, only one recent paper has reported that chitosans showed potential to disturb biofilm formation by QS inhibition and to inhibit *Chromobacterium violaceum* violacein production [25]. In two parallel studies conducted in our laboratories [26,27], we have documented the QS-inhibiting effect of both chitosan oil-core nanocapsules and chitosan-based crosslinked matrix nanoparticles. In the present work, complementary to the two parallel ones, we have investigated the antibacterial activity of 10 chitosan samples of varying *M_w_* and DA. This enabled us to select one non-toxic chitosan to explore in further detail its anti-QS activity using the *Escherichia coli* Top10 reporter strain that we have used in previous works [26,28,29,30].

## 2. Materials and Methods

### 2.1. Materials

**Scheme 1:**
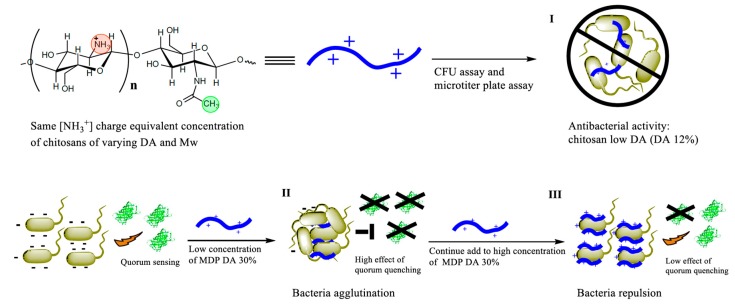
Experimental approach used to screen the chitosans with highest antibacterial activity, namely chitosans of low, medium or high degree of polymerization (DP) and low degree of acetylation (DA, 12%) (Step I). A non-antibacterial chitosan termed MDP DA 30 with medium degree of polymerization (MDP) and an experimental DA of 40% was selected for quorum sensing inhibition activity and agglutination assays at low (Step II) or high (Step III) concentrations. CFU: colony forming unit.

The parent chitosan with a molecular weight of 288 kDa and DA of 16% was purchased from Mahtani Chitosan Pvt. Ltd (Veraval, India; brand nam: Chitosan 132; batch No. SCCF 20140609). The chitosan samples were all derived from the same parent chitosan. They were depolymerized and, if needed, re-acetylated before purification. This work was conducted in our laboratory according to the methods described in our previous study [31]. The DA was determined by proton nuclear magnetic resonance spectroscopy (^1^H NMR) by dissolving the chitosans in deuterium oxide (D_2_O) with deuterium chloride (DCl) (at pD 3–4) according to the method by Lavertu et al. [32]. The molecular weight distribution and corresponding *M_w_*, *M_n_*, and polydispersity index (*I_p_*) parameters were determined via HPSEC-MALLS-DRI (Polymer Standards Service GmbH (PSS), Mainz, Germany) using an Agilent 1200 system with isocratic pump and Novema columns (30, 3000, and 3000 Å and guard column; I.D.: 8 mm; PSS) coupled online with a refractive index detector (Agilent 1200 RID, Santa Rosa, CA, USA) and multi-angle-laser-light-scattering (SLD 7000 MALLS, Brookhaven Instruments, Warwick, UK) equipped with a 5 mW He/Ne laser operating at λ = 632.8 nm. The degassed mobile phase consisted of ammonium acetate 0.2 M/acetic acid 0.15 M buffer pH 4.5 at a flow rate of 0.7 mL/min at 35 °C. Data was evaluated using the software WinGPC 7.0.1 (PSS). Chitosans with *M_w_* from ~185 to ~288, from ~85 to ~115, and from ~29 to ~58 kDa were regarded here as of high (HDP), medium (MDP), or low degree of polymerization (LDP), respectively. The rest of the identification code was obtained from the DA value (e.g., HDP DA12, etc.). *N*-(3-oxohexanoyl)-l-homoserine lactone (3OC_6_HSL) and all other chemicals were of analytical grade and purchased from Sigma (Sigma-Aldrich, Hamburg, Germany), unless stated otherwise. The overall methodological approach adopted in this work is illustrated in Scheme 1.

### 2.2. Chitosan Sample Preparation

The chitosan samples in powder form were fully dissolved in aqueous 5% stoichiometric excess of hydrochloric acid by gentle magnetic stirring (~10 h). In order to test the antibacterial activity of different chitosan samples, the same concentration of amino groups of chitosan was prepared and applied in this assay. The final concentration of amino groups was defined as 0.389 or 0.778 mM for all samples. However, the mass concentrations of samples were different according to the DA as shown in Table 1.

### 2.3. Bacterial Strains

*Escherichia coli* Top10 was transformed with the plasmid pSB1A3-BBa_T9002, carrying the BBa_T9002 genetic device (Registry of Standard Biological Parts: http://parts.igem.org/Part:BBa_T9002), kindly donated by Prof. Anderson Lab (UC Berkeley, CA, USA). The sequence BBa_T9002 was introduced by chemical transformation (Invitrogen, Life Technologies Co, Glasgow, UK) and stored as a 30% glycerol stock at −80 °C. The transformed strain is a biosensor that can respond to the 3OC6HSL and is the same strain used in accompanying studies [33]. The sequence BBa_T9002 comprises the transcription factor (LuxR) under the control of the lux pR promoter from *Vibrio fischeri*, which is constitutively expressed but is active only in the presence of the exogenous cell–cell signaling molecule 3OC6HSL.

### 2.4. Growth Media and Conditions

Bacterial strains were cultivated in Luria-Bertani (LB) and M9 minimal medium purchased from Becton, Dickinson & Co, Kehl, Germany. Ten milliliters of LB broth supplemented with 200 µg/mL ampicillin was inoculated with a single colony from a freshly streaked plate of Top10 containing BBa_T9002 and incubated for 18 h at 37 °C, shaking at 100 rpm. Each culture was then diluted 1:1000 into 20 mL M9 minimal medium supplemented with 0.2% casamino acids and 1 mM thiamine hydrochloride plus 200 µg/mL ampicillin (AppliChem GmbH, Darmstadt, Germany). The culture was maintained under the same conditions until the optical density measured at 600 nm (OD_600_) reached 0.15 (~5 h). Then 500 µL overnight culture and 500 µL 30% sterile glycerol were mixed together in cryotubes and stored at −80 °C. Before the biosensor assay was conducted, 40 µL bacteria from the glycerol stock vials was cultivated in 20 mL M9 medium plus 200 µg/mL ampicillin until the OD_600_ reached 0.04~0.07 (~4 h).

### 2.5. Assays for the Determination of Antibacterial Activity

The antibacterial activity of the chitosans was tested by two different methods. The first one consisted in recording the time evolution of the absorbance intensity (λ = 600 nm, OD_600_) during the incubation of *E. coli* in culture medium. Briefly, bacteria were cultivated in M9 medium to reach an OD_600_ of ~0.04–0.07. In a flat-bottomed 96-well-plate (Greiner Bio-One International GmbH, Kremsmünster, Austria), a 180 µL aliquot of *E. coli* culture was mixed with 20 µL of the different chitosan solutions with final equimolar amine group concentrations of 0.389 mM. As the control, 20 µL of the chitosan solutions were mixed with 180 µL M9 medium. Three different blanks were also prepared, namely 180 µL *E. coli* mixed with 20 µL water as a negative control, 180 µL M9 medium with 20 µL water to measure the absorbance background, and 180 µL *E. coli* supplied with 20 µL kanamycin (AppliChem, 0.5 mg/mL) as a positive control. The OD_600_ of the microtiter plate was measured with a Safire Tecan-F129013 Microplate Reader (Tecan, Crailsheim, Germany) every 158 s for 60 cycles (2:38 h total time) at 37 °C. Afterwards, aliquots of 20 µL were sampled from the microplate wells and remixed with fresh M9 medium to be subjected to the same measurement cycles again. Data was processed with XFLUOR4 version 4.51 and corrected by subtracting the corresponding chitosan control and the difference between the bacterial blank and the medium blank. The growth rate of controls and samples was obtained and analyzed from the original data. Using a log-linear model to regress OD_600_ (log phase) with respect to time (0–100 min), the growth rate of OD_600_ was obtained directly from the slope of the estimated regression equation. Relative data was expressed as the ratio of each measurement to the corresponding control.

The second assay aimed to estimate the number of colony forming units (CFUs) of *E. coli* under the influence of chitosans. To this end, a culture was prepared by mixing 20 mL LB medium, 20 µL ampicillin (200 µg/mL, AppliChem), and 40 µL of *E. coli* glycerol stock and incubating it overnight at 37 °C with shaking at 100 rpm. The OD_600_ was adjusted to ~0.1 using LB medium, and 180 µL of the diluted *E. coli* culture was incubated with 20 µL of each chitosan solution of identical equivalent charge concentration (0.389 or 0.778 mM) in a 96-well-plate at 37 °C and 100 rpm for 1 h. Afterwards, serial dilution was performed with 180 µL M9 medium. Then, all the dilutions were plated on LB-ampicillin-agar-plates by adding a drop of 10 µL. Plates were incubated overnight at 37 °C and colonies were counted the next day. A weighted average was calculated from the dilutions.

### 2.6. *Escherichia coli* Top10 Biosensor Assay

The 3OC_6_HSL was dissolved in acetonitrile to a stock concentration of 100 mM and stored at 20 °C. Prior to each experiment, serial dilutions from the stock solution were prepared in water to produce solutions with a concentration ranging from 100 mM to 10 nM. For a typical biosensor assay, 40 μL of the bacterial glycerol stock were cultivated with 20 mL M9 medium supplemented with 200 μg/mL ampicillin up to an OD_600_ of 0.04 to 0.07 (~4 h). Bacterial growth was monitored by measuring the optical density at λ = 600 nm (OD_600_) on a Microplate Reader Safire F129013 (Tecan, Crailsheim, Germany).

We then mixed 10 µL 3OC_6_HSL solution with 10 µL of the MDP DA 42% chitosan at varying concentrations (1.25, 2.5, 4.17, 8.33, 12.5, 25, and 50 µg/mL) in the wells of a flat-bottomed 96-well plate (Greiner Bio-One, cat. # M3061) and each well was filled with 180 µL aliquots of the bacterial culture to test for QS inhibition activity. Several controls were set up. Blank 1 wells contained 180 µL of M9 medium and 20 µL of milliQ water to measure the absorbance background. Blank 2 wells contained 180 µL of bacterial culture and 20 µL of milliQ water to measure the absorbance background corrected for the cells. Finally, positive control wells contained 10 µL of water plus 10 µL 3OC_6_HSL solution and 180 µL of bacterial culture to measure the fluorescence background. To remove any contribution of 3OC_6_HSL itself to the OD_600_ and fluorescence intensity measurements, another control consisted of 10 µL 3OC_6_HSL solution with 10 µL of the samples in the wells and each well was then filled with 180 µL M9 medium.

The plates were incubated in a Safire Tecan-F129013 Microplate Reader (Tecan) at 37 °C and fluorescence measurements (λ_ex_ = 480 nm and λ_em_ = 510 nm, 40 µs, 10 flashes, gain 100, top fluorescence) were conducted every 6 min, along with absorbance measurements (OD_600_, λ = 600 nm absorbance filter, 10 flashes) and shaking (5 s, orbital shaking, high speed). For each experiment, the fluorescence intensity (FI) and OD_600_ data was corrected by subtracting the values of absorbance and fluorescence backgrounds.

### 2.7. Scanning Electron Microscopy

A 5 mL bacterial suspension (OD_600_ = 0.2) was mixed with different concentrations of MDP DA30 chitosan (2.95, 5.88, 8.79, 28.7, and 226.6 µg/mL). The mixture was incubated at 4 °C and 100 rpm shaking for one hour. Subsequently, each mixture was filtered through a polycarbonate membrane with 1.2 μm track-etched pores mounted onto a funnel assembly connected to a vacuum pump. After filtering, the material was fixed in 2.5% aqueous glutaraldehyde for 1 h; then the membranes were washed with PBS once for 10 min after fixing. Finally, the samples were dehydrated through a graded series of aqueous ethanol solutions (10, 30, 50, 70, 90, and 100% *v*/*v*) for 10 min of each and kept in a sealed desiccator until complete dehydration. All samples were sputter-coated with gold and imaged using a scanning electron microscope (SEM, S-3000N, Hitachi, Tokyo, Japan). Micrographs were recorded digitally.

### 2.8. Statistical Analysis

Statistical analysis was carried out using Prism v6.0c (GraphPad Software Inc., La Jolla, CA, USA). All experiments were performed in triplicates to validate reproducibility and the *P* values were calculated statistically by Student’s *t*-test. Values were expressed as mean ± standard deviation (SD). Comparison analyses were performed between tests and controls.

## 3. Results

The ten chitosans with varying *M_w_* and DA investigated in this study (Table 1) were part of the series of samples that we have addressed in the frame of the Nano3Bio EU project [34]. Both DA and *M_w_* of the samples span a very wide range. This range of DA and *M_w_* envelops well the most common properties of commercial chitosans. In Table 1, one column gives the concentration (µg/mL) of each chitosan corresponding with a constant equivalent molar charge [NH_3_^+^] concentration of 0.389 mM. We have performed the antibacterial activity assays by controlling the charge-equivalent concentration, rather than the mass per volume one. Of note, two chitosan samples were not included in the experimental evaluation of their DA, namely LDP DA 20 and LDP DA 30. However, in Table 1, we have added a column with the estimated DA, as calculated from the stoichiometry of the *N*-acetylation reaction. Hence, the experimental DA values of these samples can be assumed to lie close to the expected ones, in keeping with the rest of experimentally measured samples. Also, we were unable to determine the *M_w_* and *I_p_* for the sample with code LDP DA 60. However, given that this sample corresponds to the same series of *N*-acetylated chitosans that were prepared by *N*-acetylation from the same depolymerized chitosan sample of low DA (1.6%), we are confident that the degree of polymerization of this material lies close to the rest of the samples in the ‘LDP’ series. Despite the fragmented data for these three samples, we decided to include them in the study.

### 3.1. Antibacterial Assay

**Figure 1:**
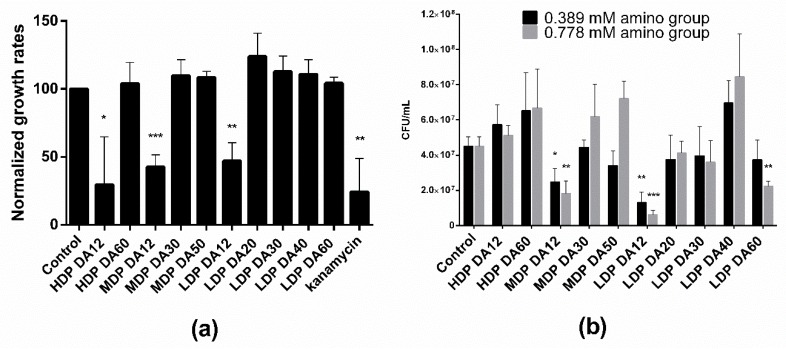
(**a**) Relative growth rate after re-seeding of *E. coli* TOP10 bacteria treated with identical charge-equivalent molar concentrations (0.389 mM) of chitosans of varying degree of acetylation and polymerization (for sample details, refer to Table 1). In the first cycle, the bacteria were incubated for 2:38 h in M9 medium at 37 °C, whereas in the second cycle, an aliquot of the bacterial culture was re-seeded in fresh M9 medium and incubated for further 2:38 h. Absorbance as measured at λ = 600 nm as a function of the chitosan concentration added to the M9 medium with *E. coli*. One (negative) control corresponds to bacteria cultivated in M9 medium, while the other (positive) control was kanamycin (50 µg/mL) added to the M9 medium. (**b**) Antibacterial effect of chitosans of varying degree of acetylation and *M_w_* with two different amino group concentrations (0.389 and 0.778 mM) on the growth of *E. coli* TOP10 incubated in a 96-well-plate at 37 °C and 100 rpm for 1 h as tested in a CFU agar plate assay. The control corresponds to untreated bacteria (* *p* < 0.05, ** *p* < 0.01 and *** *p* < 0.001; values represent average ± standard deviation (SD), *n* ≥ 3; three technical replicates).

Two distinct methods were used to investigate the antibacterial activity of the different chitosans, namely by measuring the growth rate during the exponential phase from the time evolution of the optical density (OD_600_), and by a plating assay to estimate the number of viable bacteria (CFU/mL). Notice in Figure 1a that bacteria treated with an equivalent equimolar charge concentration (0.389 mM) of the different chitosans subjected to a first incubation cycle in M9 culture medium were able to grow (albeit with differences) upon re-seeding and incubated for a second cycle. This is taken as demonstrated diagnostic evidence of a predominant bacteriostatic, rather than bactericidal, effect for all tested chitosans. Of note though, regardless of the *M_w_*, only chitosans with DA ~12% significantly reduced the relative growth rate by ~50%. In turn, the agar gel plating assay (Figure 1b) revealed that exclusively MDP and LDP chitosans of low DA (DA = 12%) reduced the number of CFUs per mL. This reduction exhibited a dose-dependent effect, as we noticed upon treatment with two different chitosan doses (0.389 and 0.778 mM). Also, LDP DA12 chitosan was more effective than MDP DA12 as revealed by the agar plate assay. Interestingly, the highest DA chitosan sample (LDP DA60, DA 69%), also reduced the plate count number in a dose-dependent manner with respect to the control. None of the other chitosans exhibited antibacterial activity, as confirmed in both assays.

### 3.2. *Escherichia coli* Top10 Quorum Sensing Biosensor Assay with Different Concentrations of Chitosan

Based on the results of the antibacterial activity assays above, we selected one of the chitosan samples that did not exhibit any effect on growth rate, namely chitosan MDP DA30 (DA 42% and *M_w_* 115 kDa), to further investigate its QS inhibition activity. To this end, we evaluated the effect of varying concentrations of this chitosan (1.25 to 50 µg/mL) on the QS response of the *E. coli* TOP10 strain. Given that the QS system operates with AHL concentrations orders of magnitude lower than those tested for chitosan in the antimicrobial assays, the concentrations of chitosan tested in this assay were 160- to 4-fold lower than those tested in the antibacterial assays. The bacterial strain is the same one that we have used in parallel studies [26,27,28,29,30,33]. It is a green fluorescent protein (GFP) reporter that does not synthesize its own AHL, namely 3OC_6_HSL, and responds by producing GFP upon exogenous addition of extremely low concentrations of 3OC_6_HSL (<1 nM).

**Figure 2:**
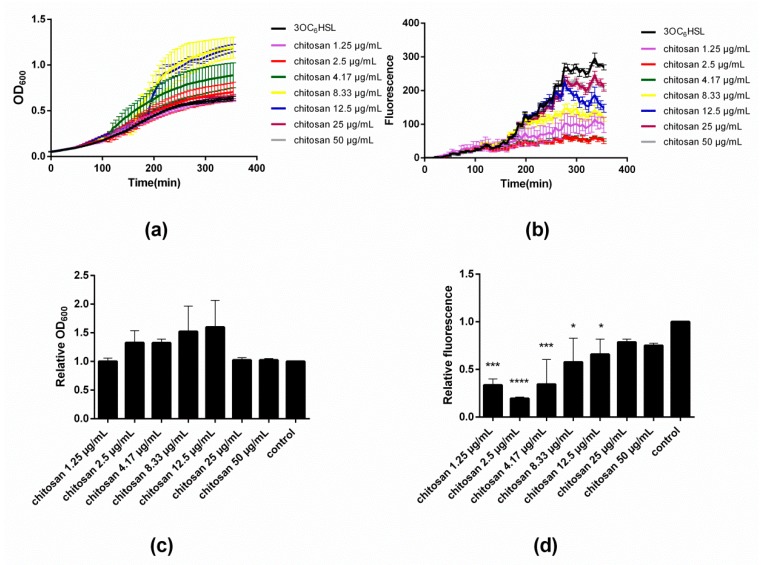
(**a**) Effect of varying concentrations of chitosan MDP DA30 (DA 42% and *M_w_* 115 kDa) on the growth of *E. coli* TOP10 as measured by assessing the absorbance at λ = 600 nm; (**b**) Effect of different concentrations of chitosan on bacterial fluorescence intensity; (**c**) Effect of varying concentrations of chitosan on endpoint OD_600_ relative to the control, corresponding with the data shown in panel a; (**d**) Effect of varying concentrations of chitosan on fluorescence/OD_600_ relative to the control, corresponding with the data shown in panel b; 3OC_6_HSL concentration: 2.5 × 10^−10^ M (* *p* < 0.05, ** *p* < 0.01, *** *p* < 0.001, and **** *p* < 0.0001; values represent average ± SD, *n* ≥ 3; three technical replicates).

**Figure 3:**
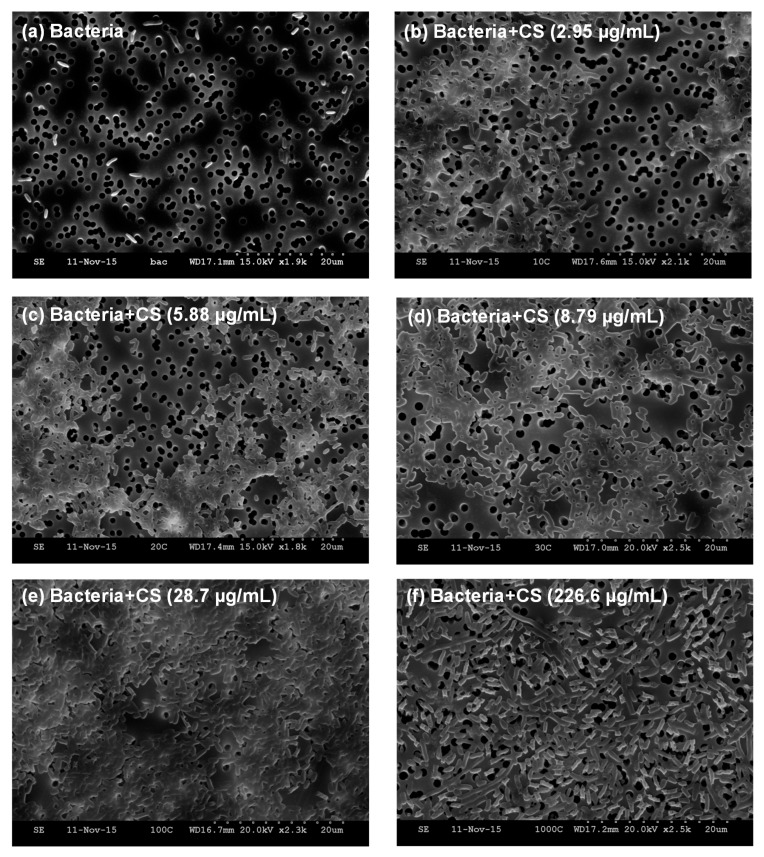
Scanning electron microscopy images of (**a**) *E. coli* TOP10 bacteria alone; and an equivalent number of bacteria (OD_600_ = 0.2) mixed with varying concentrations of MDP DA30 chitosan, namely (**b**) 2.95 µg/mL; (**c**) 5.88 µg/mL; (**d**) 8.79 µg/mL; (**e**) 28.7 µg/mL; (**f**) 226.6 µg/mL, after incubation at 4 °C and slight shaking for 1 h, supported on track-etched polycarbonate membranes. Under these imaging conditions, bacteria appear as bright rod objects. Polycarbonate support appears as a gray background, and sharply-defined track-etched pores appear as dark circles of ~1.2 µm in diameter.

Figure 2a,c illustrate the influence of varying concentrations of chitosan on the evolution and end-point values of OD_600_, respectively. Close inspection of Figure 2a reveals that the addition of chitosan in low doses of 1.25 and 2.5 µg/mL does not modify the growth curve with respect to the control. However, as the concentration of chitosan increases to 4.17 µg/mL, a sudden rise in OD_600_ occurs at time point ~110 min. The same effect occurs also with 8.33 and 12.5 µg/mL of chitosan, though at later onset times, namely ~150 and ~180 min, respectively. Upon addition of even greater concentrations of chitosan (25 and 50 µg/mL), the rise in OD_600_ is no longer appreciated and the traces mirror those of the control. The results after 350 min (‘end point’) summarized in Figure 2c illustrate that up to a concentration of 12.5 µg/mL, chitosan leads to an increase of the final OD_600_ values. Beyond this concentration, there is no effect on the bacterial growth. Figure 2b depicts the fluorescence intensity data corresponding to the same time points of the OD_600_ measurements during the microtiter plate assay. A detailed inspection of the fluorescence intensity traces reveals a rather peculiar dependence of the GFP expression on the concentration of added chitosan. At low concentrations of chitosan, namely 1.25–4.17 µg/mL, the fluorescence intensity is severely attenuated after ~120 min, and attains a minimum value at 2.5–4.17 µg/mL. At greater concentrations of chitosan (≥8.33 µg/mL), the intensity increases until almost the same value as the untreated control. Figure 3d shows the fluorescence intensity end-point values that clearly summarize the results. Notice that the minimal fluorescence intensity was assessed at a chitosan concentration of 2.5 µg/mL.

### 3.3. Scanning Electron Microscopy Imaging of *Escherichia coli* Top10 Mixed with Different Concentrations of Chitosan

With the aim of gleaning understanding regarding the observed effects of chitosan on the *E. coli* TOP10 strain, we recorded SEM images after incubating a fixed number of bacteria (OD_600_ = 0.2) with varying concentrations of MDP DA30 chitosan (2.95, 5.88, 8.79, 28.7, and 226.6 µg/mL). Figure 3a shows the image of *E. coli* TOP10 bacteria alone that appear as isolated rods. However, upon addition of chitosan at concentrations ≤28.7 µg/mL, bacteria invariably appear aggregated in conglomerates (Figure 3b–e). At the highest concentration of chitosan (226.6 µg/mL) though, bacteria again appear as isolated entities (Figure 3f). Visual inspection of the bacterial suspensions upon addition of chitosan, also reflected that the turbidity of the suspensions was maximal between 8.79 and 28.7 µg/mL (see Figure S1).

## 4. Discussion

The actual mode of action whereby chitosan exerts its antimicrobial activity is not fully understood yet. In the case of antibacterial activity, several hypotheses have been put forward to explain the mechanisms at play. One is related to the ability of the available NH_3_^+^ groups in chitosan to bind to negatively charged proteins of the outer membrane of Gram-negative bacteria leading to imbalances of the internal osmotic equilibrium and/or leakage of proteinaceous and other intracellular constituents [17,18]. Another proposed mechanism argues that chitosan penetrates the cell membrane and binds to DNA or mRNA in the cytosol, thus interfering with protein synthesis [4,5,9,35]. It has also been proposed that a chitosan’s mode of action would involve chelating trace elements leading to growth and metabolic arrest [5]. The antimicrobial activity of chitosan is known to depend on several factors either associated to the type of microorganism, the characteristics of the cell envelope, to intrinsic features of chitosan (e.g., DA, *M_w_*, chelating capacity and hydrophilic/hydrophobic characteristics), or to environmental factors (e.g., pH of the medium, temperature, time) [9,15].

In an attempt to examine in detail the influence of the charge density of chitosan (i.e., given directly by the DA), in the present work, we applied the same [NH_3_^+^] charge-equivalent concentration of chitosans of varying DA and *M_w_*. Notice that in these series of experiments to assess identical charge-equivalent concentrations, the greater the DA, the greater was the added mass concentration (Table 1); and yet only the chitosans of lower DA (i.e., dosed at the lowest mass concentrations) exhibited antibacterial activity, as evident by the growth rate in liquid culture and in the agar plate colony number counts. Regarding the *M_w_*, in the case of growth rate studies, the three chitosans of low DA LDP DA12, MDP DA12, and HDP DA12 had a strong effect on suppressing growth. However, in the plating assays, carried out in agar gels, only the medium and low *M_w_* chitosans reduced the number of CFU/mL.

A possible explanation to the apparently discrepant results between the two assays, based purely on physical grounds, could be differences in the diffusion rates of chitosan when dissolved either in the liquid or in the agar gel media for the growth rate and plating assays, respectively. In the former case, the chitosans in solution are able to diffuse freely and interact with bacteria, whereas in the agar plate assay, presumably only the chitosans of low *M_w_* were able to diffuse through the agar’s dense molecular gel network and interact with bacteria, while the high *M_w_* was not. This is consistent with the fact that LDP DA12 chitosan, of the lowest *M_w_*, had stronger activity than that of MDP DA 12. Also, these results are in overall agreement with previous studies documenting that chitosans of low *M_w_* exhibit stronger antibacterial activity due to their greater mobility and amenability to interact with the cell membrane surface [4,35]. This could be explained as the consequence of stronger electrostatic interactions between charged NH_3_^+^ groups in deacetylated d-glucosamine and the bacterial cell membrane.

In previous studies, it has been suggested that the low antibacterial effect of chitosans of high DA is the result of greater inter-chain interactions due to H-bonds in addition to the hydrophobic character of the acetyl group in *N*-acetyl glucosamine, thus leading to a more densely overlapping coiled conformation. Those interactions have been suggested to inhibit the high DA chitosan binding to the bacterial cell walls [2,7]. We noticed that chitosan LDP DA 60 (DA 69%) also reduced the plate count number in a dose-dependent manner. However, this same chitosan had no effect on growth rate in the liquid suspension assay. We do not have a convincing explanation for this result at present, particularly because the other chitosans of high DA, namely LDP DA 30 (DA 30% *M_w_* 37 kDa) and LDP DA 40 (DA 44% *M_w_* 58 kDa), did not reduce the number of viable bacteria.

Contrary to our expectations, only the chitosan of lower DA had a consistent effect on arresting the bacterial growth, even when the bacteria were treated with identical doses of charge-equivalent chitosan samples. From this experiment, it stands to reason that not only the effective amount of charged NH_3_^+^ groups in the respective chitosan is what determines the antibacterial effect of chitosan, but that other structural features are also at play. Otherwise, a similar antibacterial activity would have been exhibited by all chitosans, when the equivalent concentration of charged NH_3_^+^ was given to the bacteria. Whether a minimum block length of contiguous deacetylated d-glucosamine residues in chitosan is needed for the interaction with the bacterial cell surface has yet to be established. This would resemble a highly cooperative binding process (i.e., a minimal number of contiguous *N*-glucosamine or *N*-acetyl-glucosamine residues needed for binding). Moreover, the pattern of acetylation (PA) that might vary from block over random to an alternating pattern should also have significant impact on biological and physicochemical activity of the specific chitosan, seeing as the distribution of the acetyl group along the chitosan chain probably also affects the chitosan conformation and the ability to attach to the bacterial membrane [2,36,37]. Ongoing studies in our laboratories are currently testing the validity of these proposals using well-characterized chitosan oligomers.

For our further QS inhibition experiments, we selected only one chitosan that had no antibacterial activity, namely sample MDP DA 30 (DA 42% and *M_w_* 115 kDa). In the QS inhibition studies, both the evolution of OD_600_ and fluorescence intensity were recorded upon treating the bacteria with varying concentrations of chitosan (1.25 to 50 µg/mL). The OD_600_ traces confirmed that this chitosan did not reduce the growth rate with respect to the control. However, sudden increases in OD_600_ were observed at three concentrations, namely 4.17, 8.33, and 12.5 µg/mL. In parallel studies [26,38,39], we have observed very similar ‘bumps’ in the growth curves (OD_600_) upon addition of an increasing number of chitosan-coated nanocapsules to the same *E. coli* strain as in this study. The appearance of such sudden ‘bumps’ observed in the growth traces can be explained as the consequence of attaining specific chitosan/bacteria ratios that result in the aggregation of bacteria, and thus in the sudden increase in OD_600_ values. Our SEM results are consistent with this explanation. In the case of chitosan-based nanocapsules [26], we have found that there is an optimal ratio of nanocapsules per bacterium at which the electrical charge of both is fully compensated (i.e., the ζ-potential is ~0). At this so-called ‘stoichiometric’ ratio, the bacteria form large conglomerates and aggregation is maximal, with the behavior dependent on material concentration and cell density. Interestingly, the ‘bumps’ were only observed for three concentration ratios. It seems that only in these cases was the optimal stoichiometric ratio hit in the course of growth. At lower or greater concentrations, this optimal ratio was not attained, either because a defect or surplus of chitosan was present.

The MDP DA30 chitosan was found to have the capacity to inhibit QS as detected in the *E. coli* TOP10 strain, particularly when dosed at concentrations of 1.25 to 4.17 µg/mL. At greater doses, the QS inhibition was less accentuated. The doses of maximal QS inhibition coincide closely with those at which the ‘bumps’ in the growth traces appear. Hence, it seems reasonable to argue that the inhibition of QS is the result of the maximum in bacterial agglomeration, which results in the retardation of the diffusion of 3OC_6_HSL into the cytosol, and hence in the attenuation of GFP expression. The SEM images recorded after incubation of bacteria with varying concentrations of chitosan show that substantial bacterial aggregation occurs when chitosan is added at low concentrations (Figure 3b–e). These results are in full agreement with the results recorded in the QS inhibition assays, namely with the occurrence of ‘bumps’ in OD_600_ growth traces and with the greatest attenuation in QS activity. However, when chitosan is added at highest concentrations, bacteria appear as isolated specimens (Figure 3f). In this case, we reasoned that the high concentration of chitosan leads to an excess of net positive charge at the bacterial surface, so as to prevent their aggregation [5].

## 5. Conclusions

Taken together, our results demonstrated that fully characterized chitosans of low DA (≤12%) have a predominantly bacteriostatic activity rather than bactericidal one. Strikingly, only chitosans with DA 12% exhibited this activity, even when all chitosans were applied at the same net molar charge-equivalent concentration. Structural features of chitosans—such as hydrogen bonding, hydrophobic interactions, and possibly cooperative binding effects—might be determinant in defining the antibacterial activity of chitosans and mechanisms at play still need further elucidation. The validity of the present findings is yet to be demonstrated for other bacterial families, including other Gram-negative but also Gram-positive species. Structural differences in the bacterial envelope at the molecular level of each are likely to be essential to the specific mode of action of different chitosans. Our work has enabled us also to identify chitosans with no antibacterial activity, even when dosed at molar charge equivalent concentrations identical to bacteriostatic chitosans of low DA. We demonstrated that one of these chitosans (DA 42% and *M_w_* 115 kDa) when dosed at low concentrations (2.5 µg/mL, i.e., ~50-fold lower concentrations than for the antimicrobial assays), has QS inhibition activity. This might be attributed to maximal bacterial agglutination observed at such low doses. By contrast, greater doses of chitosan lead to attenuation of the QS inhibition effect. We are fully aware that the present work did not address the inhibition of bacterial biofilm formation, which might be also at play in controlling bacterial aggregation, driven by secretion of exo-polysaccharides and other virulent traits under QS regulation. This could be the subject of future studies.

We anticipate a high practical relevance to these findings, particularly in guiding the choice and dose of a given chitosan for applications—such as in food, wine making, water treatment, and downstream processing, among others—which are yet to be fully realized.

## Figures and Tables

**Table 1:** Characteristics of chitosan samples.

Chitosan Name	*M_w_* (kDa) ^1^	*I_p_* ^1^	Calculated DA (%) ^2^	Experimental DA (%) ^3^	Chitosan Concentration in Antibacterial Bioassays (µg/mL) ^4^
LDP DA12	29	1.6	12	12	73.5
LDP DA20	35	2.1	20	n.a	82.5
LDP DA30	37	1.7	30	n.a	96.7
LDP DA40	58	2	40	44	115.6
LDP DA60	n.a	n.a	60	69	181.7
MDP DA12	85	1.6	12	14	73.5
MDP DA30	115	1.7	30	42	96.7
MDP DA50	91	2.5	50	n.a	142.0
HDP DA12	288	2.1	12	16	73.5
HDP DA60	185	1.9	60	68	181.7

^1^ Weight average molecular weight (*M_w_)* and polydispersity index (*I_p_* = *M_w_*/*M_n_*) values as determined by high performance size exclusion chromatography with multi-angle laser light scattering and differential refractive index detection (HPSEC-MALLS-DRI); ^2^ degree of acetylation (%) values as estimated from the stoichiometry of the *N*-acetylation reaction; ^3^ experimental DA (%) values as determined by proton nuclear magnetic resonance spectroscopy (^1^H NMR); ^4^ concentration (µg/mL) equivalent to 0.389 mM [-NH_3_^+^] molar charge; n.a. = data not available.

## Supplementary Materials

The following are available online at http://www.mdpi.com/2218-273X/8/3/87/s1.

## References

[1] Raafat D., von Bargen K., Haas A., Sahl H.G. (2008). Insights into the Mode of Action of Chitosan as an Antibacterial Compound. Appl. Environ. Microbiol..

[2] Rinaudo M. (2006). Chitin and chitosan: Properties and applications. Prog. Polym. Sci..

[3] Tokura S., Ueno K., Miyazaki S., Nishi N., Kamachi M., Nakamura A. (1997). Molecular Weight Dependent Antimicrobial Activity by Chitosan. New Macromolecular Architecture and Functions.

[4] Benhabiles M.S., Salah R., Lounici H., Drouiche N., Goosen M.F.A., Mameri N. (2012). Antibacterial activity of chitin, chitosan and its oligomers prepared from shrimp shell waste. Food Hydrocoll..

[5] Rabea E.I., Badawy M.E.-T., Stevens C.V., Smagghe G., Steurbaut W. (2003). Chitosan as antimicrobial agent: Applications and mode of action. Biomacromolecules.

[6] Kong M., Chen X., Xing K., Park H.J. (2010). Antimicrobial properties of chitosan and mode of action: A state of the art review. Int. J. Food Microbiol..

[7] Goy R.C., Morais S.T.B., Assis O.B.G. (2016). Evaluation of the antimicrobial activity of chitosan and its quaternized derivative on *E. coli* and *S. aureus* growth. Rev. Bras. Farmacogn..

[8] No H.K., Park N.Y., Lee S.H., Meyers S.P. (2002). Antibacterial activity of chitosans and chitosan oligomers with different molecular weights. Int. J. Food Microbiol..

[9] Devlieghere F., Vermeulen A., Debevere J. (2004). Chitosan: Antimicrobial activity, interactions with food components and applicability as a coating on fruit and vegetables. Food Microbiol..

[10] Azuma K., Osaki T., Minami S., Okamoto Y. (2015). Anticancer and anti-inflammatory properties of chitin and chitosan oligosaccharides. J. Funct. Biomater..

[11] Sogias I.A., Williams A.C., Khutoryanskiy V.V. (2008). Why is Chitosan Mucoadhesive?. Biomacromolecules.

[12] Menchicchi B., Fuenzalida J.P., Bobbili K.B., Hensel A., Swamy M.J., Goycoolea F.M. (2014). Structure of Chitosan determines its interactions with mucin. Biomacromolecules.

[13] Nagamoto T., Hattori Y., Takayama K., Maitani Y. (2004). Novel chitosan particles and chitosan-coated emulsions inducing immune response via intranasal vaccine delivery. Pharm. Res..

[14] Roy K., Mao H.Q., Huang S.K., Leong K.W. (1999). Oral gene delivery with chitosan–DNA nanoparticles generates immunologic protection in a murine model of peanut allergy. Nat. Med..

[15] Verlee A., Mincke S., Stevens C.V. (2017). Recently developments in antibacterial and antifungal chitosan and its derivatives. Carbohydr. Polym..

[16] Luis E., Paz C.D., Resin A., Howard K.A., Sutherland D.S., Wejse P.L. (2011). Antibacterial effect of chitosan nanoparticles in *Streptococcus mutans* biofilms. Appl. Environ. Microbiol..

[17] Jeon S.J., Oh M., Yeo W.-S., Galvão K.N., Jeong K.C. (2014). Underlying mechanism of antimicrobial activity of chitosan microparticles and implications for the treatment of infectious diseases. PLoS ONE.

[18] Helander I.M., Nurmiaho-Lassila E.-L., Ahvenainen R., Rhoades J., Roller S. (2001). Chitosan disrupts the barrier properties of the outer membrane of Gram-negative bacteria. Int. J. Food Microbiol..

[19] Qi L., Xu Z., Jiang X., Hu C., Zou X. (2004). Preparation and antibacterial activity of chitosan nanoparticles. Carbohydr. Res..

[20] Ouattara B., Simard R.E., Piette G., Begin A., Holley R.A. (2000). Inhibition of surface spoilage bacteria in processed meats by application of antimicrobial films prepared with chitosan. Int. J. Food Microbiol..

[21] Blair J.M.A., Webber M.A., Baylay A.J., Ogbolu D.O., Piddock L.J.V. (2014). Molecular mechanisms of antibiotic resistance. Nat. Rev. Microbiol..

[22] Fuqua C., Parsek M.R., Greenberg E.P. (2001). Regulation of gene expression by cell to cell communication: Acyl-homoserine lactone quorum sensing. Annu. Rev. Genet..

[23] Waters C.M., Bassler B.L. (2005). Quorum sensing: Cell-to-cell communication in bacteria. Annu. Rev. Cell Dev. Biol..

[24] Kociolek M.G. (2009). Quorum-sensing inhibitors and biofilms. Antiinfect. Agents Med. Chem..

[25] Costa E.M., Silva S., Pina C., Tavaria F.K., Pintado M. (2014). Antimicrobial Effect of Chitosan against Periodontal Pathogens Biofilms. SOJ Microbiol. Infect. Dis..

[26] Qin X., Engwer C., Desai S., Vila-Sanjurjo C., Goycoolea F.M. (2017). An investigation of the interactions between a bacterial quorum-sensing biosensor and chitosan-based nanoparticles. Colloid Surf. B.

[27] Omwenga E.O., Hensel A., Shitandi A., Goycoolea F.M. (2018). Chitosan nanoencapsulation of flavonoids enhances their quorum sensing and biofilm formation inhibitory activities against an *E. coli* Top 10 biosensor. Colloid Surf. B.

[28] Qin X., Kräft T., Goycoolea F.M. (2018). Chitosan encapsulation modulates the effect of trans-cinnamaldehyde on AHL-regulated quorum sensing activity. Colloid Surf. B.

[29] Hao N.T., Goycoolea F.M. (2017). Chitosan/cyclodextrin/TPP nanoparticles loaded with quercetin as novel quorum sensing inhibitors. Molecules.

[30] Omwenga E.O., Hensel A., Pereira S., Shitandi A.A., Goycoolea F.M. (2017). Antiquorum sensing, antibiofilm formation and cytotoxicity activity of commonly used medicinal plants by inhabitants of Borabu sub-county, Nyamira County, Kenya. PLoS ONE.

[31] Santos-Carballal B., Aaldering L.J., Ritzefeld M., Pereira S., Sewald N., Moerschbacher B.M., Götte M., Goycoolea F.M. (2015). Physicochemical and biological characterization of chitosan-microRNA nanocomplexes for gene delivery to MCF-7 breast cancer cells. Sci. Rep..

[32] Lavertu M., Xia Z., Serreqi A.N., Berrada M., Rodrigues A., Wang D., Buschmann M.D., Gupta A. (2003). A validated ^1^H NMR method for the determination of the degree of deacetylation of chitosan. J. Pharm. Biomed. Anal..

[33] Vila-Sanjurjo C., Engwer C., Qin X., Hembach L., Verdia-Cotelo T., Remunan-Lopez C., Vila-Sanjurjo A., Goycoolea F.M. (2016). A single intracellular protein governs the critical transition from an individual to a coordinated population response during quorum sensing: Origins of primordial language. bioRxiv.

[34] Nano3Bio.eu. www.nano3bio.eu.

[35] Goy R.C., de Britto D., Assis O.B.G. (2009). A review of the antimicrobial activity of chitosan. Polímeros.

[36] Weinhold M.X., Sauvageau J.C.M., Kumirska J., Thöming J. (2009). Studies on acetylation patterns of different chitosan preparations. Carbohydr. Polym..

[37] Kumirska J., Weinhold M.X., Steudte S., Thöming J., Brzozowski K., Stepnowski P. (2009). Determination of the pattern of acetylation of chitosan samples: Comparison of evaluation methods and some validation parameters. Int. J. Biol. Macromol..

[38] Vila-Sanjurjo C., Hembach L., Netzer J., Remuñán-López C., Vila-Sanjurjo A., Goycoolea F.M. Covalently and ionically, dually crosslinked chitosan nanoparticles block quorum sensing and affect cell growth on a cell-density dependent manner.

[39] Vila-Sanjurjo C., David L., Remuñán-López C., Vila-Sanjurjo A., Goycoolea F.M. Bacterial quorum quenching by sub-lethal levels of chitosan nanoparticles.

