# Supplementary material for: Assessment of the Quorum Sensing Inhibition Activity of a Non-Toxic Chitosan in an N-Acyl Homoserine Lactone (AHL)-Based Escherichia coli Biosensor
Source: Biomolecules. 2018 Sep 4;8(3):87. doi: 10.3390/biom8030087 (PMC6164843; doi:10.3390/biom8030087)

biomolecules

Article

Assessment of the Quorum Sensing Inhibition Activity of a Non-Toxic Chitosan in an AHL-Based *E. coli* Biosensor

Xiaofei Qin [1], Jana Emich [1] and Francisco M. Goycoolea [1,2,*]

[1] Institute of Plant Biology and Biotechnology, University of Münster, Schlossplatz 8, 48143 Münster, Germany; qxf200459420@163.com
[2] School of Food Science and Nutrition, University of Leeds, LS2 9JT, Leeds, United Kingdom
* Correspondence: F.M.Goycoolea@leeds.ac.uk; Tel.: +44 113 343 1412

Supporting Information

Figure S1. Appearance of *E. coli* Top 10 bacteria suspensions in M9 medium alone and in the presence of varying concentrations of chitosan (as shown in labels).

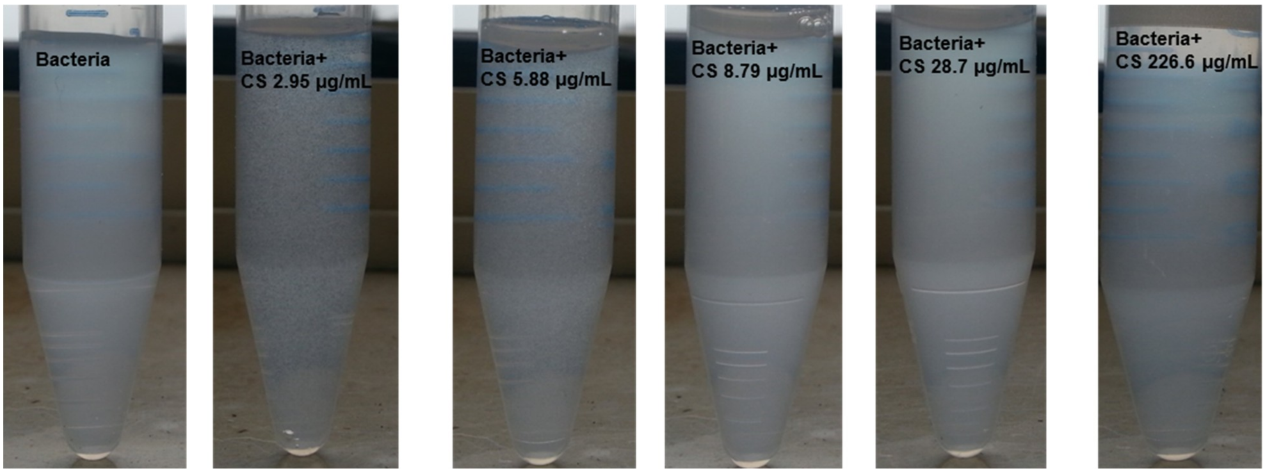

Supplement: Supplementary file 1 [file biomolecules-08-00087-s001.pdf]
